# Assessing the information desire of patients with advanced cancer by providing information with a decision aid, which is evaluated in a randomized trial: a study protocol

**DOI:** 10.1186/1472-6947-11-9

**Published:** 2011-02-14

**Authors:** Linda JM Oostendorp, Petronella B Ottevanger, Winette TA van der Graaf, Peep FM Stalmeier

**Affiliations:** 1 Department of Epidemiology, Biostatistics and HTA, Radboud University Nijmegen Medical Centre (RUNMC), Nijmegen, the Netherlands; 2 Department of Medical Oncology, Radboud University Nijmegen Medical Centre (RUNMC), Nijmegen, the Netherlands; 3 Department of Radiation Oncology, Radboud University Nijmegen Medical Centre (RUNMC), Nijmegen, the Netherlands

## Abstract

**Background:**

There is a continuing debate on the desirability of informing patients with cancer and thereby involving them in treatment decisions. On the one hand, information uptake may be hampered, and additional stress could be inflicted by involving these patients. On the other hand, even patients with advanced cancer desire information on risks and prognosis. To settle the debate, a decision aid will be developed and presented to patients with advanced disease at the point of decision making. The aid is used to assess the amount of information desired. Factors related to information desire are explored, as well as the ability of the medical oncologist to judge the patient's information desire. The effects of the information on patient well-being are assessed by comparing the decision aid group with a usual care group.

**Methods/Design:**

This study is a randomized controlled trial of patients with advanced colorectal, breast, or ovarian cancer who have started treatment with first-line palliative chemotherapy. The trial will consist of 100 patients in the decision aid group and 70 patients in the usual care group. To collect complete data of 170 patients, 246 patients will be approached for the study. Patients will complete a baseline questionnaire on sociodemographic data, well-being measures, and psychological measures, believed to predict information desire. The medical oncologist will judge the patient's information desire. After disease progression is diagnosed, the medical oncologist offers the choice between second-line palliative chemotherapy plus best supportive care (BSC) and BSC alone. Randomization will take place to determine whether patients will receive usual care (n = 70) or usual care and the decision aid (n = 100). The aid offers information about the potential risks and benefits of both treatment options, in terms of adverse events, tumour response, and survival. Patients decide for each item whether they desire the information or not. Two follow-up questionnaires will evaluate the effect of the decision aid.

**Discussion:**

This study attempts to settle the debate on the desirability of informing patients with cancer. In contrast to several earlier studies, we will actually deliver information on treatment options to patients at the point of decision making.

**Trial registration:**

Netherlands Trial Register (NTR): NTR1113

## Background

Good clinical practice encompasses optimal providing of information to patients. However, as yet the debate about the desirability of informing patients with cancer and thereby involving them in their own care process has not been resolved. On the one hand, it has been shown that patients with more severe disease may become emotionally unstable and tend to leave decision making to their physician [1-3]. Furthermore, patients with cancer may sometimes deny their illness [4]. In short, patients may wish 'not to know'. All these factors hamper the uptake of information. It is conceivable that additional stress could be inflicted upon patients by involving them in decisions which are complex, emotionally hot, with high personal stakes and have to be taken under time pressure. These circumstances are unfavourable for rational decision making [5]. Taken these arguments together, the positive effects of informing cancer patients with relevant information for treatment decision making can be questioned.

On the other hand, cancer patients desire to be informed about risks and prognosis. A systematic review of Gaston *et al*. (2005) [6] showed that almost all cancer patients wish to be fully informed, regardless of the stage of their disease. Studies using the questionnaire that was developed by Cassileth *et al*. 1980 [7] showed that 83%-92% of advanced cancer patients wanted as much information as possible, whether good or bad [7-10]. Hagerty *et al*. 2004 [11] more specifically explored the desire for prognostic information and showed that over 80% of advanced cancer patients wanted to know the longest time to live with treatment, 5-year survival rates, and average survival. Unlike the aforementioned studies that probed preferences, Elit *et al*. [12] actually delivered information to patients with advanced gynaecological cancer. A decision instrument was used to present information on two chemotherapeutic treatment options and their potential risks and benefits. Survival information was desired by and provided to 92% of the women. However, these patients were asked to make a hypothetical decision, since the treatment choice would not actually be carried out. In the present study, we will assess patients' information desire by offering information in a decision aid at the point of decision making. The treatment choice of patients will actually be carried out.

The amount of information desired may be predicted by a number of patient-related and disease-related factors. It is known that a higher amount of information is preferred by patients who are younger, better educated, have received the diagnosis more recently, and are in a less advanced stage of the disease [7,9,10,13]. In our study, where we actually deliver information using a decision aid, we will examine how various sociodemographic, medical, and psychological characteristics of the patient are related to the patient's information desire.

Current practice of information giving by physicians was investigated by studies in Australia [14,15] and the Netherlands [16], by audio taping the first consult of incurable cancer patients with their oncologists. In both Australia and the Netherlands, the majority of patients were informed about the absence of cure (75% and 84%, respectively). However, information about life expectancy or prognosis was communicated to only 58% and 39% of Australian and Dutch patients, respectively. Only half of the patients in both countries were informed about the alternative to active treatment, best supportive care (BSC). When BSC was mentioned in the Netherlands, half of the time it was mentioned in a single sentence. Obviously, not all information issues were covered in every first consultation. The oncologists may have intuitively tailored the amount and content of information to the individual patient. However, whereas studies have indicated that over 80% of patients desire all information, the percentages of Australian and Dutch patients receiving all information were noticeably lower. This raises the question whether the oncologists may have underestimated the amount of information desired by their patients. In the present study, we will assess whether medical oncologists are able to judge their patients' information desire.

A main concern of physicians is that the provision of prognostic information to seriously ill patients may provoke patient anxiety. Our target population of patients with incurable disease may be particularly anxiety-prone. To address this issue, the effects of the decision aid are evaluated by comparing the decision aid group to a usual care group. The evaluation will examine effects on patient well-being, information and decision related outcomes, and treatment choice.

In conclusion, we aim to settle the debate on the desirability of informing patients with cancer by developing a decision aid to assess the amount of information desired by patients with advanced cancer at the point of decision making. The study population will consist of patients with advanced colorectal, breast, or ovarian cancer who are treated with first-line chemotherapy and upon disease progression will face the choice between two equivalent treatment options. These patients can decide to have second-line chemotherapy in combination with BSC, or they can refrain from further chemotherapy and opt for BSC alone. Second-line chemotherapy may induce tumour response and prolong survival, but also entails a risk of serious adverse events. Our research questions are: 1a) Do these patients want to be informed about the treatment options, and specifically about their prognosis? 1b) Which factors determine whether or not these patients want to be informed? 2) Can the medical oncologist judge whether or not the patient wants the risk information? 3) What is the effect of the decision aid on patient outcomes (well-being, information and decision related outcomes, treatment choice) compared to usual care?

## Methods/Design

### Patients

In 11 hospitals in the Netherlands, all patients with advanced colorectal, breast, or ovarian cancer who have started treatment with first-line palliative chemotherapy and upon disease progression will be faced by the choice regarding second-line palliative chemotherapy will be included. Exclusion criteria are labile personality structure, as assessed by the physicians, a Karnofsky performance score lower than 60, and insufficient knowledge of the Dutch language. The study has been approved by the research ethics committees of all participating hospitals.

### Procedure

The medical oncologist, or a nurse who first consulted the medical oncologist, will inform eligible patients in a general way that this study focuses on 'how to involve the opinion of patients in the treatment'. Next, he or she will ask whether the researcher can contact the patient by phone about this study. The oncologist or nurse is instructed not to discuss that survival information can be provided in the study, in order not to lose patients not desiring such information. After the visit of the patient, the oncologist fills in the medical history on the inclusion form. He or she also makes a judgment whether or not the patient will desire information about adverse events, tumour response, and median survival that will be offered in the interview (research question 2). In the phone call, patients are explained that data are collected by means of three questionnaires and possibly an interview. All patients who agree to participate will provide written informed consent.

Patients will be sent a baseline questionnaire (t1) to collect sociodemographic data, well-being measures, and measures that predict information desire. When tumour progression is diagnosed, second-line chemotherapy will be offered by the oncologist and randomization will be performed. Patients in the usual care group will receive information about the treatment choice from their oncologist. In the intervention group, patients will receive the same information, and will in addition be offered information in an interview using a decision aid. At the moment of disease progression, the medical oncologist is asked to estimate patient survival, because this is expected to be associated with the patient's information desire.

**Figure 1:**
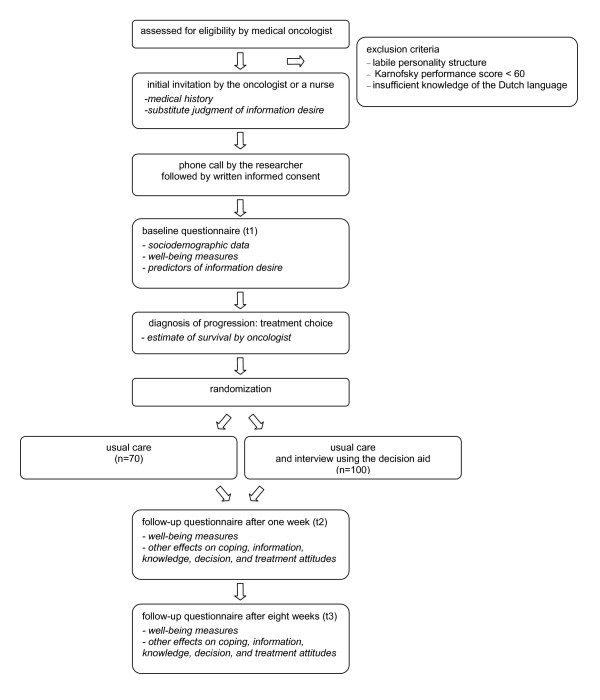
Overview of study procedure

To evaluate the effect of the decision aid, two follow-up questionnaires will be sent to the patients, one week (t2) and eight weeks (t3) after the treatment choice, before the first evaluation of chemotherapy. Figure 1 displays the complete procedure.

### Randomization

Randomization will be performed to determine whether the patient will receive usual care (n = 70) or usual care plus the decision aid (n = 100). To accomplish this, a computer generated randomization list is prepared that is stratified by hospital, using a block size of 3. Each participating hospital will receive three sets of sealed envelopes, related to colorectal, breast, and ovarian cancer patients. When disease progression is diagnosed by the oncologist, a nurse will perform the randomization by opening the appropriate envelope.

### Development of the decision aids

Decision aids on second-line treatment options for patients with advanced colorectal, breast, and ovarian cancer are developed. In this decision aid, the two treatment options are explained. Next, an overview of the potential risks and benefits of the two treatment options is presented. The information on risks and benefits is obtained from systematic reviews of the literature for each tumour type. Risk information includes the occurrence of serious adverse events, which are defined as grade 3 or 4 according to the Common Terminology Criteria for Adverse Events v3.0 (CTCAE) and are presented as adverse events that can be life threatening, can result in a hospital admission or prolongation of existing hospitalization, or can lead to a persistent or significant disability/incapacity. Tumour response is defined according to WHO or RECIST criteria and classified into overall response (complete or partial response), stable disease, and progressive disease. For both serious adverse events and tumour response, the probability that outcomes occur is presented in frequencies (n of 100 patients) and by means of pie charts, using a mixed frame, e.g. '22 out of 100 patients will experience severe diarrhoea, 78 out of 100 patients will not experience severe diarrhoea'. Information on survival is presented in the format of median survival.

### Interview

The decision aid is delivered by a nurse or by the researcher. First, the two treatment options are explained. Then, to familiarize patients with the general issue of trade-offs between benefits and risks, an example of a risky two-attribute non-cancer related choice is presented. After this example, the interviewer explains the first item of the decision aid regarding serious adverse events. This explanation includes the definition of a serious adverse event and gives the patient insight in the type of information that can be expected. Next, the patient is asked whether or not the information on serious adverse events is desired. The same procedure is followed for the other items on tumour response and survival. At the end of the interview, the patient can take home a brochure with information, tailored to the information desire of the patient. The interviewer fills in a questionnaire to register information desire and to provide a short evaluation of the interview.

### Outcome measures

Table 1 shows an overview of all outcome measures and the moment of measurement.

**Table 1:** Outcome measures

Subject		Questionnaire	Inclusion	T1	Disease progression	Interview	T2	T3
**Sociodemographic characteristics**				x				
**Previous chemotherapy**				x				

**OUTCOME MEASURES & PREDICTORS OF INFORMATION DESIRE**								

**Well-being**	General Health			x			x	x
	Anxiety & Depression	Hospital Anxiety and Depression Scale		x			x	x
	Cancer Worries	Adapted Lerman's Cancer Worry Scale		x			x	x
	Health-related quality of life	EORTC QLQ-C15 PAL		x			x	x

**Coping**	Helpless/Hopeless, Avoidance, Fighting Spirit	Mental Adjustment to Cancer Scale		x			x	x
	Decision Styles	Michigan Assessment of Decision Style		x				
	Participation Preferences	Problem-Solving Decision-Making Scale		x				
	Perceived participation	Problem-Solving Decision-Making Scale					x	x
	Perceived involvement	Whelan					x	x
	Death avoidance	Death Avoidance Scale		x				

**Information**	Preference for information			x				
	Amount of information			x			x	x
	Information from oncologist						x	
	Undesired information						x	
	Information desire					x		
	Satisfaction with quality of information						x	x
	Balanced presentation of information						x	
	Evaluation of information						x	x
	Numeracy	Subjective Numeracy Scale		x				

**Knowledge**	General subjective knowledge			x				
	Treatment subjective knowledge						x	
	Objective knowledge						x	
	Subjective risk						x	
	Objective risk						x	

**Decision**	Decision satisfaction-uncertainty	Decision evaluation scale					x	x
	Decision control	Decision evaluation scale					x	x
	Weighing pros and cons	Decision evaluation scale					x	x
	Treatment choice					x	x	
	Strength of treatment preference					x	x	

**Treatment attitudes**	Valuations						x	x
	Treatment satisfaction							x
	Preferences for Quality/Quantity of Life	QQ Questionnaire		x				

**PHYSICIAN'S QUESTIONS**								

**Medical history**	Tumour and treatment characteristics		x					
**Substitute judgment**	Substitute judgment of information desire		x					
**Prognosis**	Estimate of survival				x			

#### Sociodemographic variables and medical history

Self-report data are collected at t1 on demographic variables (age, marital status, having (grand)children, being religious, working status, and education) and on previous chemotherapy, including experienced benefits and adverse events, and time since last chemotherapy. Tumour and treatment characteristics such as primary tumour site, previous chemotherapy, and time since diagnosis are filled in on the inclusion form by the medical oncologist. At the moment of disease progression, the medical oncologist is asked to estimate patient survival. Answer possibilities are 3-6 months, 6-9 months, 9-12 months, and more than 12 months.

#### Well-being

Patients rate their general health in the previous week on an 11-point rating scale (0-10). Anxiety and depression are assessed with the Hospital Anxiety and Depression Scale (HADS) [17]. Cancer worries are rated with three questions, adapted from Lerman *et al*. [18] and Stefanek *et al*. [19]: 'Did you think of cancer last week?', 'Did these thoughts affect your mood?', and 'Did these thoughts affect your daily activities?'. Health-related quality of life is assessed by means of the EORTC QLQ-C15 PAL quality of life questionnaire for cancer patients in palliative care [20].

#### Coping

Coping with cancer is assessed with the Mental Adjustment to Cancer scale [21]. Three coping strategies are assessed: helplessness/hopelessness, avoidance, and fighting spirit. The patient's decision style is assessed with The Michigan Assessment of Decision Style (MADS) [22]. The MADS covers (1) avoidance (four items, e.g. 'I prefer not knowing the possibility that unexpected things could happen to me'); (2) deferring responsibility (three items, e.g. 'I would follow the recommendations of my physician'); (3) information seeking (four items, e.g. 'I would spend as much time as I could gathering information'); and (4) deliberation (five items, e.g. 'I would carefully consider the risks of each option as I was making a choice'). The general participation preference at baseline is measured with the two decision-making items from the Problem-Solving Decision-Making Scale [23]. The questions are as follows: 'When the risks and benefits of the treatment options are known to you, (1) who decides how acceptable those risks and benefits are for you and (2) who decides what treatment should be selected?'. Answers range from 'only the physician' to 'only me'. Perceived participation is assessed by the same questions in past tense, after the decision is made. Perceived involvement is measured by asking patients whether they felt they were offered a choice between BSC plus chemotherapy and BSC alone [24]. In addition, patients are asked whether their opinion regarding the treatment mattered. Both questions use a yes/no response. Death avoidance will be measured by means of the Death Avoidance Scale, which was adapted by Kaplowitz *et al*. 2002 [25] from the original Death Acceptance Scale developed by Klug and Sinha (1987) [26]. The two items are as follows: 'I avoid discussing death when the occasion presents itself' and 'I make a conscious effort to avoid dwelling on thoughts of death', and are measured on a 5-point scale (1 = strongly disagree, 5 = strongly agree).

#### Information

The preferred amount of information is measured on a 11-point scale ranging from 0 (I want to know nothing about the illness and its treatment) to 10 (I want to know everything there is to know about the illness and its treatment) [8]. The amount of information received about the treatment choice is measured on a 7-point scale ranging from 1 (I received way too little information), 4 (I received exactly enough information), to 7 (I received way too much information). Information received during the consultation with the oncologist, regarding adverse events, tumour response, and survival, is assessed using a yes/no response. Whether patients received any undesired information is asked with a question using a yes/no response. The key variable information desire is described under 'Interview' above. Satisfaction with the quality of information is asked with three questions related to information on adverse events, tumour response, and survival. Responses are on a 6-point scale ranging from 1 (not satisfied) to 6 (very much satisfied). The balanced presentation of information is evaluated on a 5-point scale from 1 (clearly in favour of chemotherapy plus BSC), 3 (balanced), to 5 (clearly in favour of BSC alone). Six questions are asked about unpleasant, shocking, frightening, and threatening experiences with the information received, measured on a 5-point scale from 1 (no negative experience) to 5 (very negative experience). Numeracy, i.e. the ability to handle basic probability concepts, needed for the interpretation of risk information, is measured by the Subjective Numeracy Scale (SNS) [27].

#### Knowledge

Patients rate their own knowledge (subjective knowledge) on cancer treatments with a single question on a 10-point scale ranging from 1 (very bad) to 10 (excellent). Two questions are asked to assess the subjective knowledge with respect to risks and benefits of treatment options on a 10-point scale. Objective knowledge will be measured with a questionnaire containing five statements related to the two options BSC plus chemotherapy and BSC alone. These statements have to be judged as right or wrong. Subjective risk perception is measured by three questions on the chance of experiencing a serious adverse event from 1 (very high) to 5 (very low), the chance that stable disease or a response will be achieved by BSC plus chemotherapy, as opposed to BSC alone from 1 (much higher) to 7 (much lower), and the chance of experiencing pain on treatment with BSC plus chemotherapy, as opposed to BSC alone from 1 (much higher) to 7 (much lower). Objective risk perception is asked for the chance of experiencing severe diarrhoea, and the chance of achieving a response. Patients are asked to give a risk estimate for these two outcomes in a range from 0% to 100%.

#### Decision

Satisfaction and uncertainty regarding the choice between BSC plus chemotherapy and BSC alone are assessed using five items [28], e.g. 'I find it hard to make this choice', 'I am satisfied with my decision', measured on a 5-point scale (1 = strongly disagree, 5 = strongly agree). Patients are also asked about their feeling of control regarding this treatment choice using five items [28], e.g. 'This decision is made without me', 'I feel pressure from others in making this choice', measured on a 5-point scale (1 = strongly disagree, 5 = strongly agree). Weighing of pros and cons of the treatment options was asked with a single question 'I weighed the pros and cons', on a 5-point scale (1 = strongly disagree, 5 = strongly agree). Treatment choice is collected by asking which treatment patients prefer. Response options are: BSC plus chemotherapy, BSC alone, don't know. Strength of treatment preference (for one or the other option) is asked on a 4-point Likert scale ranging from 1 (weak preference) to 4 (very strong preference).

#### Treatment attitudes

Valuations for each of the two treatment options are asked on a 10-point scale ranging from 1 (very bad) to 10 (excellent). Satisfaction with the received treatment and with the physical and emotional effect of treatment is measured on a 6-point scale from 1 (dissatisfied) to 6 (very satisfied). Patient's attitudes towards striving for length (quantity) or quality of life are assessed with 8 items by the 'QQ Questionnaire' [29]. High scores on the quantity or quality scale indicate the importance of length and quality of life, respectively.

#### Substitute judgment

When the medical oncologist informs patients about this study, he or she is asked to judge whether or not the patient will desire information about adverse events, tumour response, and median survival that will be offered in the interview.

### Sample size calculation

Our primary research question is whether or not patients want to be informed. For study question 1a, about 100 patients in the intervention group will suffice to estimate an assumed desire rate for prognostic information of 80% with 95% confidence interval from 71% to 87%. For study question 1b, regarding factors that determine information desire, again about 100 patients will suffice to detect a weak correlation of 0.28 between a predictor and information desire, with α = 0.05 and a power equal to 0.82. For question 2, dealing with the ability of the medical oncologist to judge whether or not the patient wants this information, the same analysis applies as for study question 1b. For study question 3, which addresses the effect of the introduction of decision support compared to usual care on patient well-being, the intervention group is compared to the usual care group. Because one of the main concerns of physicians is that information on prognosis provokes anxiety, anxiety is used as the main outcome measure. We assume that the HADS anxiety difference between the two groups at t3 is equal to 2.2, and the correlation between the measurements at t1 and t3 equals 0.72. The standard deviation in each cell is about 6, corresponding to a medium sized effect size of 0.36. With α = 0.05, and 70 patients in each group, the power to detect the assumed effect is equal to 0.81. In conclusion, 100 patients in the intervention group and 70 patients in the usual care group suffice to answer the above research questions. Assuming that 30% of the patients refuse to give informed consent, and that 1% of patients has died at t3, 246 patients will have to be approached.

### Statistical Analysis

In all the analyses, scale values will be calculated only if at least half of the items are available, by imputing the mean of the available items. Question 1a about the information desire will be answered by calculating the desire rate for prognostic information with a 95% confidence interval. For question 1b, which factors determine information desire, patients will be grouped according to information desire, ranging from 0 (no information desired) to 3 (information on serious adverse events, tumour response, and survival desired). Then comparisons between these four groups will be made. For categorical variables, the Chi-square test will be used. Continuous data will also be analyzed using the Chi-Square test, after a median-split subdivision into two categories. The HADS scores will be divided by use of a clinical cut-off point of 8 [17,30,31]. Bivariate analyses will be presented with p-value of variables related with information desire at the level of p < 0.2. Finally, these variables will be entered simultaneously in a regression model.

For question 2, agreement between the substitute judgment of the oncologist and the patient information desire is determined. Agreement can arise from chance; a measure correcting for chance agreement is the κ statistic, ranging from 0 to 1. A κ of 0.2, 0.5, and 0.8 indicates poor, moderate, and good agreement, respectively [32,33]. In bivariate and multivariate analyses, associations will be sought between agreement and patient variables (sociodemographic, medical, psychological, knowledge and information). Dichotomized variables associated with agreement at a level of p < 0.2 will be entered simultaneously in a regression model.

Question 3, which addresses the effect of the introduction of decision support compared to usual care on patient well-being, will be answered by comparing the intervention group with the usual care group. Analyses of covariance corrected for differences at baseline are used to test the effect of the intervention. For group comparisons at a single point in time, a t test will be performed for continuous variables and the Chi-square test for categorical variables.

### Ethical considerations

Ethical approval for the study is obtained from the regional ethics review committee (CMO Arnhem-Nijmegen) and from the research ethics committees of all participating hospitals. All participating patients will sign an informed consent form.

## Discussion

This study attempts to settle the debate on the desirability of informing patients with cancer. To this end, the amount of information desired by patients with advanced cancer is investigated by offering information in a decision aid. Furthermore, the study will show whether it is possible to predict the amount of information desired, either by patient and disease-related factors, or by judgment of the medical oncologist. Concerns that the information may provoke patient anxiety are addressed by evaluating the effects of the decision aid using a randomized controlled design.

The study design has some limitations. Selection bias could be introduced when oncologists select patients who are more open to information or are in a better psychological state. When the data collection is completed, the role of selection bias can be investigated by comparing the resulting sample to other study samples, and comparing patient characteristics between physicians that included few versus many patients. Selective patient participation based on patients' information desire is addressed by informing eligible patients about the study in a general way, not mentioning the provision of (prognostic) information.

Patients have to fill in a baseline questionnaire that includes predictors for information desire and a baseline measurement for the effect of the information. Therefore, it is not feasible to include patients at the moment of the treatment choice; patients have to be included during first-line chemotherapy. This timing may positively affect the inclusion rate, since patients will be less distressed during first-line chemotherapy than at the moment of disease progression. On the other hand, due to the interval between the baseline questionnaire and the interview, the association between information desire and predictors may be weakened. In addition, the correlation between the effect measures at baseline (t1) and follow-up (t2 and t3) may be weakened.

Randomization is, for practical reasons, performed by using sealed envelopes. In some of the 11 participating hospitals, there will be little time between the diagnosis of disease progression and subsequent consultation with a nurse. Therefore, instant randomization will be required and envelopes are a very practical method to perform this. The use of envelopes leaves a possibility to subvert the randomization procedure. However, a plain attempt to allocate a patient out of sequence will be discovered because the envelopes are sequentially numbered and the allocation can be compared with the predetermined sequence. The oncologist could also decide not to randomize a patient, but this would not go unnoticed since all patients are registered on the trial before the moment of randomization, therefore we will be able to compare patients who were and were not randomized.

Blinding of the medical oncologists and the patients is not feasible in this type of research, because patients may want to discuss the information from the decision aid with their oncologist. However, patients are blinded to the intervention in that they are not aware of the exact content of the decision aid; they are only informed that a new method of information giving is investigated.

A major strength of this study is that, in contrast to many previous studies, we will actually deliver information on treatment options to patients at the point of decision making. The results of this study can be applied to improve the provision of information in daily clinical practice. Generalization to the target population is facilitated by the broad inclusion criteria that are used in this study and the use of the decision aid by various nurses in the 11 participating hospitals. Furthermore, the method used to assess patients' information desire very much resembles information giving in clinical practice, by actually providing information at the moment of the treatment choice.

## Competing interests

The authors declare that they have no competing interests.

## Authors' contributions

PS, PO and WG conceived of the study and developed the research protocol. LO drafted this manuscript, which was commented on by PS, PO, and WG. All authors read and approved the final manuscript.

## Pre-publication history

The pre-publication history for this paper can be accessed here:

http://www.biomedcentral.com/1472-6947/11/9/prepub
